# Comparative Analysis of Methods for Somatic Cell Counting in Cow’s Milk and Relationship between Somatic Cell Count and Occurrence of Intramammary Bacteria

**DOI:** 10.3390/vetsci10070468

**Published:** 2023-07-17

**Authors:** Vladimír Hisira, František Zigo, Marián Kadaši, Róbert Klein, Zuzana Farkašová, Mária Vargová, Pavol Mudroň

**Affiliations:** 1 Clinic of Ruminants, University of Veterinary Medicine and Pharmacy, 04181 Košice, Slovakia; vladimir.hisira@uvlf.sk (V.H.); marian.kadasi@uvlf.sk (M.K.); robert.klein@uvlf.sk (R.K.); pavol.mudron@uvlf.sk (P.M.); 2 Department of Nutrition and Animal Husbandry, University of Veterinary Medicine and Pharmacy, 04181 Košice, Slovakia; zuzana.farkasova@uvlf.sk; 3 Department of the Environment, Veterinary Legislation and Economy, University of Veterinary Medicine and Pharmacy, 04181 Košice, Slovakia; maria.vargova@uvlf.sk

**Keywords:** mastitis, indirect method, pathogen

## Abstract

**Simple Summary:**

Reducing the incidence of mastitis is one of the primary objectives of dairy farms. Currently, new rapid diagnostic methods, both direct (culture) and indirect, are being introduced; these methods serve to detect the number of somatic cells in milk. This study was conducted to compare them; in the study, three indirect methods were tested (the California Mastitis Test, the Porta somatic cell count milk test, and the DeLaval cell counter). Their agreement, sensitivity, and specificity were analyzed with laboratory somatic cell count (SCC) measurements. The highest agreement (83.1%) and sensitivity (81.0%) were recorded with the CMT, and the highest specificity (97.5%) was determined with the DeLaval cell counter. When comparing the agreement of elevated SCCs, the highest value of tested farm methods with bacterial findings was found with the Porta SCC (73.7%). Based on the given results, it can be concluded that all these tests can be used in the rapid diagnosis of subclinical mastitis since each test has certain advantages over the others.

**Abstract:**

The aim of this study was to compare three on-farm commercial methods for the indirect detection of subclinical mastitis in dairy cows: the California mastitis test (CMT), the Porta side somatic cell count milk test (Porta SCC), and the DeLaval cell counter (DCC), with the Fossomatic cell count (FSCC), and to evaluate the relationship between the determined somatic cell count SCC and the occurrence of intramammary pathogens in the milk of dairy cows. A total of 284 sensory unchanged mixed milk samples, collected during the milking on a dairy farm, were analyzed in this study for somatic cell counts by the mentioned on-farm tests. Quarter milk samples (*n* = 583) from all the selected cows were cultured. The agreement, sensitivity, and specificity of the three indirect commercial diagnostic tests (the CMT, the Porta SCC, and the DeLaval cell counter) were calculated, and the FSCC was used as the gold standard. The results were analyzed statistically using the Pearson correlation test and the paired *t*-test. The CMT matched with the FSCC in 83.1% of the samples, with the Porta SCC in 80.6%, and with the DCC in 80.3% of the samples. The sensitivity and specificity reached 81.0% and 92.9% for the CMT, 79.4% and 90.7% for the Porta SCC, and 75.8% and 97.5% for the DCC, respectively. The correlation between the FSCC and the Porta SCC was 0.86 (*p* < 0.0001), and between the FSCC and the DCC, it was 0.92 (*p* < 0.0001). The differences between them were insignificant. Bacteria were detected in 130 (22.3%) quarter milk samples. The most prevalent bacteria were *Enterococcus* spp. (36.2%), followed by *E. coli* (20%), coagulase-negative staphylococci (13.1%), *A. viridans* (9.2%), *Streptococcus* spp. (9.2%), *Proteus* spp. (6.2%), and *S. intermedius* (3.9%). Contagious isolates (*S. aureus*) were detected in 3 quarter milk samples (2.3%). The agreement between the individual tests and the microbiological culture was as follows: 69.2% for the CMT; 73.7% for the Porta SCC; 71.6% for the DCC; and 76.5% for the FSCC. Higher SCCs were detected in the milk samples contaminated with bacteria than in the healthy milk (*p* < 0.001). No significance was found between the presence of individual species of intramammary pathogens and the different levels of SCCs. Based on the results, bacteria are the predominant cause of subclinical mastitis. The increased SCC of some milk samples with no presence of bacteria meant that the increase could have been caused by numerous other agents (viruses, fungi, or algae) or factors for mastitis in the dairy industry.

## 1. Introduction

Despite the considerable efforts made by many scientists and experts in mastitis, the disease has not been eliminated on dairy farms and remains a serious problem, causing significant losses to farmers. According to the available literature, the prevalence of mastitis varies significantly between countries, regions, and farms worldwide [1,2,3,4]. The occurrence of mastitis is a complex of interactions between the host, the environment, and the etiological agent, and is influenced by several factors [5]. To date, many indirect methods (tests and devices) have been developed for the rapid detection of SCC levels, the principle of which is based on the interaction of somatic cell DNA and the relevant reagents. The somatic cell count (SCC) is one of the basic indicators of milk quality assessment; in accordance with this parameter, the standard SCC of ≤400,000 SC/mL for class 1 milk is currently applied in Slovakia [6]. This standard also applies to other EU countries, as well as to Australia, New Zealand, and Canada. For the USA, the standard is ≤750,000 SC/mL [7], while a count less than 500,000 SC/mL is applied in Brazil and India [8,9]. The acceptability break point for the SCC is 2 × 10^5^ cells/mL in Sweden [10]. When such levels are exceeded, dairy cows are diagnosed with subclinical mastitis. The milk quality decreases and leads to economic losses on dairy farms; these losses are mainly caused by a decrease in milk yield [11]. The reduction in milk yield is approximately 2.5% at 100,000 cells increase above the value of 200,000 SC/mL, and therefore the highest milk yield is given by dairy cows with an SCC up to 200,000 [12].

In dairy cows with an SCC from 200,000 to 400,000 SC/mL, the milk production differs by 4.6%, with the loss amounting to 372 kg of milk, and for dairy cows with an SCC of over 400,000 SC/mL, the loss is 11.9% (959 kg). In terms of monetary loss per cow, this represents USD 107 per dairy cow with an SCC between 200,000 and 400,000 SC/mL, and USD 275 per dairy cow with an SCC above 400,000 SC/mL. If indicators such as cow culling and calf mortality and morbidity were included in the final losses caused by subclinical mastitis, these losses would be higher. According to studies presented in the National Mastitis Council Report [13], the decrease in milk production affected by SCCs of up to 500,000 SC/mL reached 6%, and it reached 18% with SCCs of up to 1,000,000 SC/mL. In another study, in the case of clinical mastitis, it was revealed that margins over feed costs were diminished by EUR 250–300 [14]. In Slovak research, the loss of milk production was 9% in SCCs of up to 500,000 SC/mL and 12% in SCCs of up to 1,000,000 SC/mL [15]. In the financial impact assessment of subclinical mastitis on Colombian dairy farms, milk production losses per median-sized farm ranged from 1.3% to 13.5%; on small farms, they ranged from 0% to 12.8%. The mean of the financial losses per 10 cows was USD 692 [16].

The prevalence of subclinical mastitis (SCM) is higher than clinical form; there are 15 to 40 cases of SCM for every clinical inflammation of the udder [17]. Thus, the overall losses associated with subclinical mastitis are higher. In another study, milk with an SCC up to 70,000 SC/mL was referred to as healthy milk, and the level of 100,000 SC/mL indicated a reduction in milk yield [18]. According to Hamann, SCCs in milk of below 100,000 SC/mL for a long period is an indication of a healthy udder [19]. The likelihood that milk is contaminated with pathogens increases with SCCs above 200,000 SC/mL [20].

The rapid diagnosis of subclinical mastitis (MS), employing not only the commonly used but also the new tests and methods, with which the number of somatic cells is determined, is very important because in the case of subclinical mastitis, the milk shows no changes and appears healthy. Therefore, the use of effective screening tests can prove to be a good investment for farmers in the long term, as they facilitate rapid diagnosis and enable the early treatment of affected cows.

The aim of this study was to compare three commercial diagnostic tests (the CMT, the Porta SCC milk test, and the DeLaval cell counter, with the results determined by the Fossomatic FC) and to compare their results, sensitivity, specificity, correlation, regression dependence, and advantages and disadvantages. The SCCs recorded by these methods were compared with the bacterial incidence determined by microbiological culture.

## 2. Materials and Methods

The monitoring took place on a dairy farm in Eastern Slovakia in the district of Kosice. Holstein dairy herds were housed on the farm in a free stall system with a manure solid bedding; from these herds, 284 cows not affected by the clinical form of mastitis were randomly selected for a comparison of the detection methods during the 3 spring months. The average milk yield was 9000 kg.

The cows were milked twice daily in a herringbone milking parlor (Agromont Nitra, Slovakia) with twelve fixation boxes in two rows opposite each other. Prefoam+ (Hypred S.A., Dinard, France) was used for udder hygiene before milking and was applied as foam. After foaming, the teats were mechanically cleaned with dry paper wipes which were intended for cleaning the entire udder and for fore-stripping. The milking vacuum was set at 42 kPa with a pulsation ratio of 60:40 at a rate of 52 c/min. The milking was automatically stopped when the milk flow dropped to 0.2 L/min. The teats were disinfected by teat dipping with HM-VIR-Film (Agromont Nitra, SK) after the milking process. The milk was stored in refrigerated milk tanks at +5 °C and removed daily at around 11.30 a.m.

### 2.1. Milk Collection

After a clinical examination of the mammary glands and physical examination of the selected milk, the visually unchanged milk samples were collected during milking (mixed samples from the whole udder of one cow) from a special container of the milking parlor in two tubes. The first was used for on-farm testing and the second for laboratory somatic cell counting.

### 2.2. Somatic Cell Counting

The SCC was determined in fresh cow’s milk using a semi-quantitative CMT, a Porta SCC milk test (Porta SCC, PortaCheck, Inc., White City, USA), and a DeLaval cell counter (DCC, DeLaval International AB, Tumba, Sweden) within 1 h after milking. The identical samples were sent to the accredited laboratory (Certified Milk Quality Assessment Centre of the Breeding Services of the Slovak Republic s.r.o. in Žilina; FSCC, Fossomatic TM FC, Denmark) for SCC analysis using the Fossomatic FC. The samples were preserved with potassium dichromate as the SCC determination was performed the following day.

#### 2.2.1. California Mastitis Test

The CMT is a commonly used rapid farm test. The principle of the test is a chemical reaction between Abeson tea (triethanolamine salt of alkylbenzene sulfonic acid) and the DNA of somatic cells in milk. Phenol red serves as a pH indicator. Using this test, the SCC was evaluated in milk according to the change in the viscosity of the milk after the addition of the reagent. The price of the analysis is 1.4 cents/sample.

#### 2.2.2. Porta SCC Milk Test

The Porta SCC milk test belongs to a group of new, commercial on-farm tests for the determination of SCCs on dairy farms. The principle of this test is a chemical reaction between the dye on the strip and the enzyme (esterase) present in the somatic cells of milk. The intensity of color in the test strip is positively correlated with the somatic cell count. The intensity of the blue color is directly proportional to the SCC in the milk. The results are evaluated 45 min after the application of the activator that triggers the chemical reaction. Using a digital reader, the exact SCC is determined based on the color change. The price of the analysis is 1.92 EUR/sample.

#### 2.2.3. DeLaval Cell Counter

The DeLaval cell counter is a battery-powered portable optical device for detecting the SCC in milk within one minute. After filling with milk, a cartridge containing a small amount of reagent (propidium iodide) is placed into the machine where it is exposed to light. The somatic cells in the milk in contact with the reagent emit fluorescent signals, and based on the fluorescence intensity, the device counts the somatic cells in the milk. The price of the analysis is 2 EUR/sample.

### 2.3. Bacteriological Examination

A microbiological culture was performed with 583 randomly selected quarter milk samples. Briefly, the milk samples (10 μL) were plated on Columbia blood (5% (*v*/*v*)) agar (OXOID Ltd., Basingstoke, Hants, UK) and incubated at 37 °C for 24–48 h. The additional tests were the Gram strain test, catalase activity, coagulase test, deoxyribonuclease test, and hemolysis test by subsequent cultivation on selective media Staphylococcal medium N° 110, Baird-Parker agar, Edwards Medium, MacConkey Agar (OXOID Ltd., Basingstoke, Hants, UK) for 18–24 h; these tests were used for the identification of staphylococci, streptococci, and enterococci. To identify bacterial strains, the commercial biochemical kits STREPTOtest 24, STAPHYtest 24, resp. ENTEROtest 24 (Erba Lachema, Brno, Czech Republic) were used. All the kits were evaluated according to an accuracy of over 90% by the software TNW ProAuto 7.0^®^ (Erba Lachema Ltd., Brno, Czech Republic).

### 2.4. Statistical Analysis

The results of the SCC were determined using a commercially manufactured CMT, the Porta SCC and a DCC device were compared with the results determined by the FSCC. The SCC measured by the Fossomatic FC was considered the gold standard. The milk was evaluated in terms of the degree of subclinical mastitis, as follows: healthy milk SCC ≤ 200,000; 200,000 to 400,000, doubtful results; up to 1,000,000, mild subclinical mastitis; 1,000,000–5,000,000, moderate SM; and over 5,000,000, severe SM. Agreement of the methods (%) was assessed as follows: Porta SCC vs. FSCC and DCC vs. FSCC—results of the SCC of up to 1 million were considered identical with a difference not greater than 100,000. In samples above 1 million, an agreement was defined when the SCC difference was not higher than 250,000. Other methods of statistical evaluation were the sensitivity and specificity tests. Correlations with the FSCC (Pearson’s coefficient) were also calculated for the Porta SCC and DCC tests.

Agreement of an increased SCC and the occurrence of bacteria in the milk were assessed by percentage. A statistical analysis was carried out in the form of a Student’s *t*-test, which was used to test the relationship between the SCC in contaminated milk and intramammary pathogens. Further to this, the relationship between the SCC and the bacterial species was analyzed by an ANOVA test.

## 3. Results

Out of the 284 analyzed milk samples (Table 1), subclinical mastitis (SM) was detected by CMT in 97 milk samples, while mild SM was detected in 31, moderate in 38, and severe in 28 milk samples. The Certified Laboratory of Breeding Services s.r.o. detected a higher number of samples with subclinical mastitis (115), of which 44 were samples with mild, 51 with moderate, and 20 with severe SM (Table 1). The average level of the SCC when measured by the Fossomatic FC instrument reached 643.9 thousand SC/mL with mild SM, 2612 mil. SC/mL with moderate SM, and 8520 mil. SC/mL with severe SM (Table 1).

The testing of the 284 samples by the Porta SCC milk test provided the following results: 96 milk samples with SM, of which there were 78 samples with a mild and moderate form and 18 samples with SCCs over the detection limit, which indicated severe SM. The DCC analyzer determined SM in 98 milk samples, of which there were 42 samples with mild and 56 with moderate SM. This method found no severe SM (as this was beyond the detection capabilities of this device) (Table 1).

**Figure 1:**
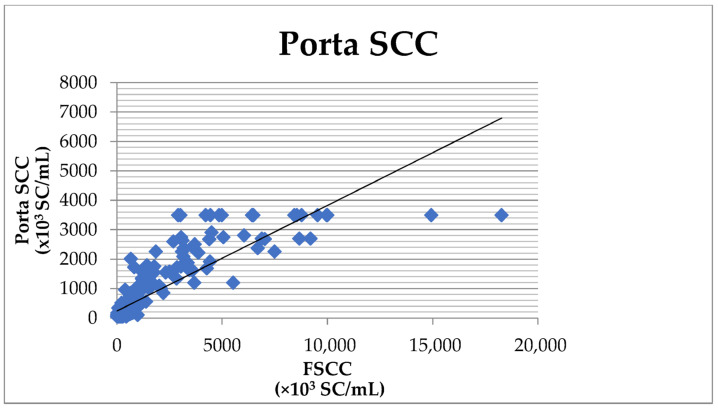
Correlation between the SCC determined by the Porta SCC milk test and the Fossomatic FC.

The comparison of the results obtained by the CMT and the FSCC showed 83.1% agreement, 81.0% sensitivity, and 92.9% specificity (Table 2). The overall agreement between the Porta SCC and the FSCC was 80.6%, with the sensitivity of the Porta SCC compared to the FSCC reaching 79.4% and the specificity reaching 90.7% (Table 2). The correlation coefficient (r) reached the value of 0.859 (Figure 1) with a high significance (*p* < 0.001).

**Figure 2:**
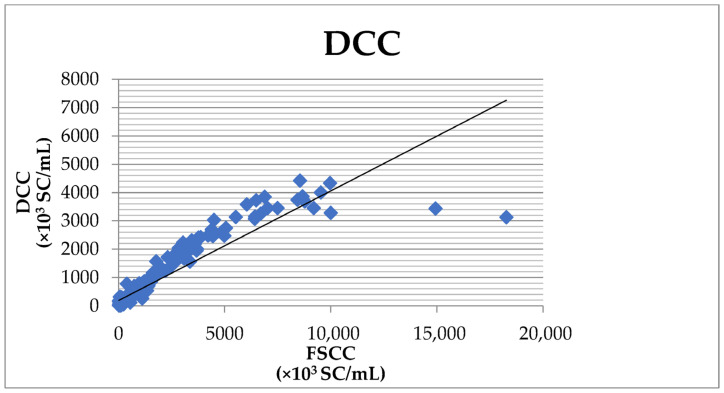
Correlation between the SCC determined by the DCC and the Fossomatic FC.

The overall agreement between the DCC and the FSCC results was 80.3%, with the sensitivity reaching 75.8% and the specificity reaching 97.5% (Table 2). The correlation coefficient of 0.917 (Figure 2) was significant at the level of *p* < 0.001.

When comparing agreement at certain somatic cell levels, the highest agreement was recorded for SCCs up to 200,000 by all the methods. Conversely, the lowest agreement between the studied methods was determined for SCCs from 400,000 to 1,000,000 SC/mL (Table 3, Table 4 and Table 5).

The most commonly used mastitis tests include the CMT. The results presented in this study indicate its high compliance, sensitivity, and specificity when used for the laboratory determination of SCCs. With such positive indicators, its additional advantages include its ease of use, favorable detection time, and price.

The Porta SCC milk test is sensitive and specific, and its compliance with the Fossomatic method exceeded 80%. Another advantage of this test is the easy handling and size of the device. The disadvantages of this assay include the lengthy 45 min enzymatic reaction after the addition of the reagent. It is necessary to adhere to this time, because if the time limit is exceeded, false positive results may occur. This test cannot be used with chilled milk. As a high milking speed is required on large-capacity farms, the use of the Porta SCC milk test is limited with respect to the required time. Another disadvantage is the detection limit of 3.5 million SC/mL in milk.

When evaluating the DCC method, several advantages and disadvantages were noted. The advantages included detection time (up to 2 min), easy handling, and good agreement with the standard. Moreover, with this device, we could determine the SCC not only in fresh milk but also in tank samples of chilled cow’s milk. The biggest disadvantage is that this device cannot provide high-accuracy readings for samples with high SCC levels, and its detection limit is 4 million SC/mL in milk.

Of the 583 randomly selected quarter samples, bacteria were detected in 130 samples (22.3%). The most prevalent bacteria were *Enterococcus* spp. (36.2%), followed by *E. coli* (20.0%), coagulase-negative staphylococci (CoNS; 13.1%), *A. viridans* (9.2%), *Streptococcus* spp. (9.2%), *Proteus* spp. (6.2%), and *S. intermedius* (3.9%; Table 6). Contagious isolates (-) were detected in 3 quarter milk samples (2.3%; Table 6).

Agreement between the CMT and the microbiological culture was noted in 69.2% of the samples, with the Porta SCC in 73.7%, and with the DCC in 71.6%; there was agreement between the FSCC and bacteriological incidence in 76.5%. In the bacteria-contaminated milk samples, a significantly higher mean SCC was found than in the healthy milk samples (*p* < 0.001), with the mean SSC in the contaminated milk analyzed by the FSCC being 3461.8 (×10^3^ SC/mL); by the DCC, it was 1549.8 (×10^3^ SC/mL); and by the Porta SCC, it was 1294.2 (×10^3^ SC/mL) (Table 7). The mean SCC of the uncontaminated milk was 253 (×10^3^ SC/mL) when counted by the FSCC, 187.4 (×10^3^ SC/mL) when counted by the DCC, and 138.8 (×10^3^ SC/mL) when determined by the Porta SCC (Table 7).

## 4. Discussion

Bovine mastitis is a problem that almost every dairy farmer encounters on a daily basis. On a world scale, this is one of the most widespread and serious diseases of dairy cattle a with significant impact on milk yield and also on the overall health of the cows. Subclinical mastitis is a particularly significant problem, and its detection is not as easy as that of clinical mastitis. The quick detection of mastitis significantly reduces economic losses on farms. Therefore, many companies have made an effort to introduce appropriate indirect diagnostic tests or devices into the market for the determination of subclinical mastitis in a simple and fast way with high accuracy. Such indirect methods include the CMT, the Porta SCC milk test, the DeLaval cell counter, and the Fossomatic FC. The CMT is one of the basic commonly used screening tests for the indirect diagnosis of subclinical mastitis. In our study, this test showed the highest agreement of the observed parameters with the laboratory SCC results determined by the Fossomatic FC. In line with our findings, the study carried out in Brazil determined that the sensitivity of the CMT in identifying subclinical mastitis was above 80%, and the specificity was above 90% [21].

By the CMT, the samples were evaluated at the moment of sampling and in the laboratory using a fluoro-opto-electronic method (Fossomatic 90). Additional monitoring of the specificity and sensitivity of the CMT was carried out with a standard culture assay, whereby the CMT sensitivity reached 75% and the specificity reached 71% [22]. According to the study by Gosvami et al. [23], the sensitivity of the indirect assays compared to the culture assay decreased in the following order: SCC (97.46%), modified CMT (69.62%), and modified white side test (63.29%). In the next research, by assessment of different diagnostic methods for subclinical mastitis high sensitivity (100% for the SCC and 96% for the CMT) was recorded [24].

In a Lithuanian experiment, several indirect methods were used to diagnose subclinical mastitis (the Mastest (developed by the Lithuanian Veterinary Institute), California mastitis test (CMT), Bernburg, Profilac reagent, and Eimü-milchtest) and were assessed. After collecting 1014 milk samples, the SCC was laboratory-determined by flow cytometry tests. In addition, electrical conductivity (EC) was measured using a Mastiii Indikaator. The electrical conductivity of the milk was increased in dairy cows with subclinical mastitis. The results of the Mastest correlated best with the number of somatic cells (95.5 to 100%). The results of the Mastest also correlated well with the other mentioned tests [25]. The analysis focused on the comparison of the effectiveness of the commonly used indirect diagnostic tests (CMT, electrical conductivity—EC, and somatic cell count—SCC) for the detection of subclinical mastitis in dairy cows; the results of the microbiological culture were considered as a gold standard. It was revealed that of the 135 milk samples tested by the CMT, EC, and the SCC, the CMT showed the highest accuracy (73.3%), but also gave the highest number of false positive reactions (24.6%) and the lowest number of false negative reactions (28.6%) [26].

In the next experiment, 364 quarter milk samples were analyzed by the CMT and the DCC. The DCC was used as a reference method for the measurement of the SCC. The optimal threshold limit of the SCC for using the CMT to identify an IMI was above 200,000 cells/mL. Based on this threshold limit, AUC was 0.87, sensitivity was 0.79, specificity was 0.95, and κ was 0.76 [27]. When we compared the SCCs in dairy milk determined by the Porta SCC and the Fossomatic cell count, we detected a correlation coefficient of 0.859, an agreement of 80.6%, a sensitivity of 79.4%, and a specificity of 90.7%. In 2004, Law reported the results of an evaluation of a Porta SCC milk test reflectometer. Based on the visual interpretation of the intensity of the color changes on indication papers, the results of the Porta SCC were compared with the results of an electronic reading. In the case of visual interpretation, the correlation was 0.63, the sensitivity was 76%, and the specificity was 94%. Using a reflectometer, the author recorded a higher correlation (r = 0.87), a sensitivity of 87%, and a specificity of 91% [28]. A high correlation was also found (r = 0.81) when comparing the results of this test with an electronic SCC measurement [29].

In other research, three Porta tests (reader, color, and quick) were analyzed for the diagnosis of subclinical mastitis (SCM) and an intramammary infection (IMI). The reference method of SCC measurement was DDC, with SCM defined as an SCC of >200,000 cells/mL. A bacterial culture of milk samples was used to diagnose an IMI based on the growth of ≥100 cfu/mL. For diagnosing SCM by the Porta tests, the Porta SCC color test was the most accurate at dry-off (AUC 0.90, Se 0.91, Sp 0.81, κ 0.71) and at freshening (AUC 0.86, Se 0.74, Sp 0.95, κ 0.72), at an optimal cut-point of ≥250,000 cells/mL, but one analysis required 45 min. The analysis time of the CMT was 2 min to produce results. A California mastitis test score was higher than the Porta SCC color test for diagnosing SCM at dry-off (AUC 0.95, Se 0.95, Sp 0.86, κ 0.81) and at freshening (AUC 0.88, Se 0.79, Sp 0.95, κ 0.76). Of all the Porta tests, the Porta SCC quick test achieved higher values for diagnosing IMI at dry-off (AUC 0.81, Se 0.81, Sp 0.78, κ 0.40) and required 5 min to produce a result, whereas the Porta SCC color test achieved the highest values for diagnosing an IMI at freshening (AUC 0.80, Se 0.75, Sp 0.79, κ 0.38) [30]. In another study, a comparison of the performance of the portable somatic cell counter (Porta SCC) and the laboratory-based somatic cell counter (Fossomatic) was assessed in the diagnosis of subclinical mastitis in the milk of buffalo. The sensitivity of the Porta SCC was 94.12% and specificity 87.30%, respectively. A substantial agreement (κ = 0.70) between the results of the 2 tests was also observed [31].

According to our testing of the DCC, the correlation coefficient of the SCC was r = 0.917 (*p* < 0.001), the agreement was 80.3%, the sensitivity was 75.8%, and the specificity was 97.5%. In a study conducted in Japan, the results of SCCs determined in milk from 99 udder quarters with subclinical mastitis by the DCC were compared with those obtained by the Fossomatic FC [32].

The SCCs determined by the Fossomatic instrument were considered the gold standard. A correlation coefficient of the SCC was r = 0.963 (*p* < 0.001) and was recorded between the results of the DeLaval cell counter and the Fossomatic FC, which is in agreement with our results. In the Czech Republic, a comparison of three methods of SCC determination (direct microscopic method (DM) of somatic cell counting, measurement by the DeLaval cell counter (DCC), and Fossomatic 90 (FSC)) in the milk of several livestock species (cows, sheep, and goats) and in human breast milk was performed, together with a correlation and regression analysis. The examination of cow’s milk showed the highest correlation between DM and the fluoro-opto-electronic (FSC) method (r = 0.99). The correlation coefficient in DM milk reached 0.99 when comparing the PM and the DCC. The correlation coefficient between the DCC and FSC for the SCC in cow’s milk was r = 0.99. When evaluating the DCC device, the authors noticed several advantages and disadvantages. The advantages included detection time (1 min), easy handling, and good compliance with the standard. Moreover, with this device, one could determine the SCC not only in fresh milk but also in tank samples of chilled cow’s milk. The biggest disadvantage was that this device could not determine the SCC with higher accuracy at high SCC levels [33].

In ovine milk, the same values of the DCC were found. The DCC used for somatic cell counting of the ovine milk samples showed high coefficients of regression (b 0.91 to 1.01) and correlation (r > 0.99) [34]. In non-lactating bovine mammary gland secretion, a comparison of commercial somatic cell counters to quantify somatic cells was measured by agreement between them and analyzed based on Lin’s concordance correlation coefficient (CCC). The quantification of somatic cells was performed using several methods (microscopically (MSCC), with a DeLaval cell counter (DSCC) and by a DHIA laboratory using a Fossomatic FC (FMSCC). The MSCC was used as a reference method. The CCC of DSCC was 0.81 and FMSCC 0.88, respectively [35].

The agreement between the tested methods and the microbiological culture was 69.2% for the CMT, 73.7% for the Porta SCC, 71.6% for the DCC, and 76.5% for the FSCC. Some scientists claimed that the first bacteria were detected at 200,000 SC/mL [36,37,38]. If this is true, then the agreement between the tests and the incidence of intramammary pathogens should be higher and, of course, the potential contamination of the milk during milking should be taken into consideration. The presence of bacteria in milk is also detected in latent mammary gland infections. Accordingly, latent udder infections in this study exceeded 20%. The high significance of the mean SCC also infected the quarter milk samples (210.5 × 10^3^) in comparison with the uninfected ones (32.72 × 10^3^) [39].

Higher mean SCCs were detected in the milk samples contaminated by bacteria compared to those of the healthy milk (*p* < 0.001). The mean SSC in contaminated milk detected by the FSCC was 3461.8 (×10^3^ SC/mL); by the DCC, it was 1549.8 (×10^3^ SC/mL); and by the Porta SCC, it was 1294.2 (×10^3^ SC/mL). In the uncontaminated milk, the SCC levels reached 253 (×10^3^ SC/mL) when detected by the FSCC, 187.4 (×10^3^ SC/mL) by the DCC, and 138.8 (×10^3^ SC/mL) by the Porta SCC (Table 7).

The somatic cell count is an important qualitative parameter of milk. It indicates the susceptibility of cows to mastitis and it is used to diagnose an intramammary infection (IMI), mostly subclinical mastitis in the dairy industry. A high SCC in milk has a negative effect on raw milk quality [40]. So far, the relationship between the SCC and an IMI has been investigated in many studies around the world [41,42]. The close relationship (*p* < 0.001) between the presence of an IMI and an increased SCC was also demonstrated in our study. Malinowski et al. [43] investigated the relationship between the SCC and the etiological agents of mastitis. The milk samples with an SCC lower than 200,000 SC/mL were mostly (59.6%) culture negative. The highest incidence of bacterial species such as *S. aureus*, *Streptococcus* spp., and Coagulase-negative staphylococci (CNS) was found between 200,000 and 2,000,000 SC/mL. In contrast to the previous study, the SCC milk of infected *S. aureus* was below 200,000 in our research. *Streptococcus* spp. and CNS were isolated in the milk samples with an SCC above 1,000,000 SC/mL.

In the previous study conducted in Poland, samples with more than 2 million/mL of SC were infected mainly with CAMP-negative and CAMP-positive streptococci and Gram-negative bacilli. The highest SCC (≥ 10 million/mL) in the foremilk samples was associated with intramammary infections caused by *Arcanobacterium pyogenes* (95.5%), *Streptococcus agalactiae* (57.6%), and Gram-negative microorganisms (46.5%). Mastitis caused by *Prototheca* sp. (64.5%), yeast-like fungi (60.2%), and *Streptococcus* sp. (55.1%) was related to very high SCCs (≥5 million/mL). An IMI caused by *S. aureus* (76.2%), CNS (84.2%), Gram-positive bacilli (72.4%), and *Corynebacterium* sp. (83.2%) increased the SCCs in a weaker manner than the previous pathogens. These levels of SCCs were less than 5 million/mL [44].

Research conducted by Djabri et al. [44] showed different SCC values for different bacterial species (×1000/mL). The SCCs of negative milk ranged from 27 to 600; from 191 to 9433 in milk contaminated with *S. aureus*; from 561 to 4758 with *Str. Agalactiae*; from 809 to 1944 with *Str. Dysgalactiae*; from 851 to 1 085 with *Str. uberis*; from 590 to 9009 with coliforms; from 90 to 3040 with CoNS; and from 128 to 1352 with *Cor. bovis*.

In a review from a South African study [45], the association between the SCC and eight intramammary pathogens was studied. The highest SCCs were found in an IMI caused by *S. dysgalactiae,* followed by *S. aureus* and *S. agalactiae.* More than 84% of *T.pyogenes* isolates are related to SCCs > 200,000 SC/mL. Some contagious bacteria were cultured in milk with an SCC < 200,000 SC/mL: *S. dysgalactiae* (4.2%), *S. aureus* (5.9%), and *S. agalactiae* (9.0%). According to the next experiment, the mean SCC of milk from healthy quarters was 39,000 SC/mL and in milk infected with CNS was ranged from 168,000 SC/mL (*S. chromogenes*) to 193,000 SC/mL *(S. hyicus*) [46]. In Finnish dairy farms, the mean SCC of milk with persistent CNS infection was over 600,000 cells/mL and about 60,000 cells/mL in healthy quarters [47].

According to a Brazilian study, the geometric means of the results of a bacteriological examination reached 52,000 in milk samples with no growth, 85,000 in samples with coagulase-negative staphylococci, 587,000 with *S. aureus*, and 432,000 with other streptococci and *S. agalactiae* (1,572,000; 333,000) [48]. In another study, the mean SCC in milk from *A. viridans*-infected cows was significantly higher (1,000,000 SC/mL) (*p* < 0.01) compared to that of healthy cows (72,400 SC/mL) [3].

The distribution of bacteria in milk with different SCCs was highly variable. Surprisingly, some intramammary pathogens could be isolated even with a very low SCC of 10,000–25,000/mL. Out of 17 such milk samples, 5 isolates of streptococci, 10 isolates of staphylococci, 5 isolates of CoNS, and 8 isolates of *E. coli* were obtained [49]. In a study carried out at the Bangladesh Agricultural University Dairy Farm and in the surrounding areas, the highest mean SCC was determined in an IMI caused by *Enterobacter* spp. (338,000 cells/mL). Lower values of the SCC were in milk infected by *Bacillus* spp. (319,200 cells/mL), CNS (268,170 cells/mL), *Staphylococcus aureus* (218,310 cells/mL), *Escherichia coli* (200,750 cells/mL), and *Pseudomonas aeruginosa* (66,330 cells/mL) [39].

## 5. Conclusions

In conclusion, the above tests were deemed to be suitable and reliable methods for somatic cell detection in cow’s milk. Thanks to its features and price, the classic CMT still maintains its very wide use. The presented results allowed us to state that, when selecting from the newly offered relevant tests, the determination of the somatic cell counts using the DeLaval cell counter device proved to be suitable, as it is able to determine SCCs not only in fresh but also in cooled milk tank samples. As for the Porta SCC milk test, it has limited use as it can only make SCC measurements in fresh milk, but the results of the compliance of this test with the Fossomatic FC were better and more accurate than those obtained with the DeLaval cell counter. Our study also confirmed that bacteria predominantly cause subclinical mastitis; this was found by the observation of a direct relationship between the high number of somatic cells in culture-positive milk samples and their presence.

## Figures and Tables

**Table 1:** Interpretation of the SCC detected by four methods.

	Fossomatic FC	DCC	Porta SCC	NK Test
	n/SCC × 10^3^/mL			
Below 50 × 10^3^/mL			69/below detection limit of the reader	
50–200 × 10^3^/mL			86/120.8	
0–200 × 10^3^/mL	118/91.7 (41.6%)	145/91.2 (50.5%)	155 (54.6%)	159
200–400 × 10^3^/mL	51/284.2 (18.0%)	41/275.6 (15.0%)	33/298.2 (11.6%)	28
400–1000 × 10^3^/mL	44/643.9 (15.5%)	42/645.3 (15.6%)	31/628.7 (10.9%)	31
1200–3500 × 10^3^/mL			47/1907.4 (16.6%)	
Over 3500 × 10^3^/mL			18/over detection limit of the reader (7.2%)	
1200–5000 × 10^3^/mL	51/2612.5 (18.0%)	56/2509.6 (18.9%)		38
over 5000 × 10^3^/mL	20/8520.6 (7.0%)	0		28
Mean SCC (×10^3^/mL)	284/1262.2	284/747.8	284/663.4	284

**Table 2:** Comparison of three methods with the FSCC.

	Agreement	Sensitivity	Specificity
CMT vs. Fossomatic FC	83.1%	81.0%	92.9%
Porta SCC vs. Fossomatic FC	80.6%	79.4%	90.7%
DeLaval CC vs. Fossomatic FC	80.3%	75.8%	97.5%

**Table 3:** Comparison of the CMT with the FSCC.

CMT vs. FSCC	Agreement	Sensitivity	Specificity
0–200,000	97.5%/115	-	97.5%
200,000–400,000	68.6%/35	77.3%	99.2%
400,000–1200 × 10^3^	61.4%/27	75.9%	97.5%
1200 × 10^3^–5000 × 10^3^	86.3%/44	91.1%	98.3%
Over 5000 × 10^3^	75.0%/15	80.0%	-

**Table 4:** Comparison of the Porta SCC milk test with the FSCC.

Porta SCC vs. FSCC	Agreement	Sensitivity	Specificity
0–200,000	95.8%/113	-	95.9%
200,000–400,000	68.6%/35	81.0%	96.7%
400,000–1200 × 10^3^	50.0%/21	69.8%	98.3%
1200 × 10^3^–5000 × 10^3^	88.2%/45	91.1%	99.2%
Over 5000 × 10^3^	65.0%/13	74.1%	-

**Table 5:** Comparison of DeLaval cell counter with FSCC.

DCC vs. FSCC	Agreement	Sensitivity	Specificity
0–200,000	98.3%/116	-	96.6%
200,000–400,000	82.4%/42	86.4%	99.2%
400,000–1200 × 10^3^	70.5%/31	77.2%	100.0%
1200 × 10^3^–5000 × 10^3^	76.5%/39	81.0%	100.0%
Over 5000 × 10^3^	0%/0	0%	-

**Table 6:** Occurrence of intramammary pathogens and the SCC in dairy cows (×10^3^ SC/mL).

Bacteria	Number of Positive Quarter Milk Samples	Porta SCC (x)	DCC (x)	FSCC (x)
Contagious pathogens	3 (2.3%)	170.0	146.0	145.0
*Staphylococcus aureus*	3			
Environmental pathogens	127 (97.7%)			
*Enterococcus* spp.	47	1357.3	1833.1	4123.8
*E. coli*	26	1318.2	1591.7	4349.8
CoNS	17	902.2	1219.0	2377.8
*S. epidermidis*	6			
*S. chromogenes*	7			
*S. warneri*	3			
*S. auricularis*	1			
*Aerococcus viridans*	12	2228.6	1678.4	3363.9
*Streptococcus* spp.	12	1900.0	2736.7	5854.8
*Proteus* spp.	8	830.0	1710.3	3078.0
*S. intermedius*	5	95.0	231.0	335.5
Total	130	1294.2	1549.8	3461.8

Note: CoNS—coagulase-negative staphylococci, x–average value.

**Table 7:** Distribution of intramammary pathogens and the SCCs in dairy cows (×10^3^ SC/mL).

	Porta SCC (x)	DCC (x)	FSCC (x )
Milk with bacterial contamination	1294.2 ***	1549.8 ***	3461.8 ***
Healthy milk	138.8 ***	187.4 ***	253 ***

Note: *** *p* < 0.001, x–average value.

## Data Availability

All existing data are listed in the manuscript.
